# Permutation Entropy-Based Interpretability of Convolutional Neural Network Models for Interictal EEG Discrimination of Subjects with Epileptic Seizures vs. Psychogenic Non-Epileptic Seizures

**DOI:** 10.3390/e24010102

**Published:** 2022-01-09

**Authors:** Michele Lo Giudice, Giuseppe Varone, Cosimo Ieracitano, Nadia Mammone, Giovanbattista Gaspare Tripodi, Edoardo Ferlazzo, Sara Gasparini, Umberto Aguglia, Francesco Carlo Morabito

**Affiliations:** 1 Department of Science Medical and Surgery, University of Catanzaro, 88100 Catanzaro, Italy; michele.logiudice@unirc.it (M.L.G.); ferlazzo@unicz.it (E.F.); s.gasparini@unicz.it (S.G.); u.aguglia@unicz.it (U.A.); 2 DIIES Department, University “Mediterranea” of Reggio Calabria, 89100 Reggio Calabria, Italy; 3 Department of Neuroscience & Imaging, University G. d’Annunzio Chieti e Pescara, 66100 Chieti, Italy; giuseppe.varone@unich.it; 4 DICEAM Department, University “Mediterranea” of Reggio Calabria, 89100 Reggio Calabria, Italy; cosimo.ieracitano@unirc.it (C.I.); nadia.mammone@unirc.it (N.M.); 5 Regional Epilepsy Center, Great Metropolitan Hospital “Bianchi-Melacrino-Morelli” of Reggio Calabria, 89124 Reggio Calabria, Italy; tripodi.giovanbattistag@gmail.com

**Keywords:** EEG, PNES, epilepsy, machine learning, deep learning, convolutional neural network, wavelet, permutation entropy, interpretability

## Abstract

The differential diagnosis of epileptic seizures (ES) and psychogenic non-epileptic seizures (PNES) may be difficult, due to the lack of distinctive clinical features. The interictal electroencephalographic (EEG) signal may also be normal in patients with ES. Innovative diagnostic tools that exploit non-linear EEG analysis and deep learning (DL) could provide important support to physicians for clinical diagnosis. In this work, 18 patients with new-onset ES (12 males, 6 females) and 18 patients with video-recorded PNES (2 males, 16 females) with normal interictal EEG at visual inspection were enrolled. None of them was taking psychotropic drugs. A convolutional neural network (CNN) scheme using DL classification was designed to classify the two categories of subjects (ES vs. PNES). The proposed architecture performs an EEG time-frequency transformation and a classification step with a CNN. The CNN was able to classify the EEG recordings of subjects with ES vs. subjects with PNES with 94.4% accuracy. CNN provided high performance in the assigned binary classification when compared to standard learning algorithms (multi-layer perceptron, support vector machine, linear discriminant analysis and quadratic discriminant analysis). In order to interpret how the CNN achieved this performance, information theoretical analysis was carried out. Specifically, the permutation entropy (PE) of the feature maps was evaluated and compared in the two classes. The achieved results, although preliminary, encourage the use of these innovative techniques to support neurologists in early diagnoses.

## 1. Introduction

Epilepsy is a chronic neurological disorder that affects the nervous system. Over 65 million cases have been reported worldwide [1]. It is caused by altered neuronal activity in the brain and is characterized by recurrent and unpredictable episodes called epileptic seizures (ES). It is ordinarily diagnosed by a neurologist along with long interviews and clinical exams. Epilepsy may be due to a brain injury, or have a genetic, immune, brain structure or metabolic cause, but unfortunately for many patients, the cause remains unknown [2].

Electroencephalography (EEG) is a measure of the electrical activity produced by the human brain, recorded on the scalp of the head. It is the most useful diagnostic tool for epilepsy [3] as it provides detailed information, with excellent temporal resolution, on the state of the brain. Therefore, EEG may be useful for discrimination between ES and psychogenic non-epileptic seizures (PNES), which in contrast, are episodes of movements, sensations or behaviors that are similar to epileptic seizures but do not have an epileptic origin and do not show any ictal epileptiform activity [4,5]. PNES typically begin in young adulthood and, these patients frequently are misdiagnosed and treated for epilepsy. Correct diagnosis is important because of the potential iatrogenic hazards, such as side effects of anti-epileptic drugs and failure to recognize pseudo-status-epilepticus with a potential outcome of intensive care unit treatment and intubation [6,7,8]. The differential diagnosis could be very complex, also because interictal EEGs in subjects with ES may be normal or non-specific using visual analysis, as usually occurs for PNES patients. On the contrary, the association of some features with a specific class of subjects could be a source of clear class determination for an artificial intelligence (AI) system. In fact, the literature [9,10,11] yields examples of EEG data processing with machine/deep learning (DL) with excellent results.

Furthermore, the use of these complex classification algorithms in critical areas has led to a growing interest in the interpretability of the results, i.e., in understanding how the networks behaved.

Permutation entropy (PE) is a tool for the analysis of time series that allows one to code important information even in time dynamics in a simple, robust way and with low computational costs. The PE can be used for the understanding of complex and chaotic systems, to provide interpretability of the behavior of time series in the classification with AI (deep) methods.

In the present study we analyzed resting state EEG recordings from subjects with ES and subjects with PNES by means of a DL pipeline including a wavelet transformation and a classification with a convolutional neural network (CNN). Experimental results proved that the proposed CNN outperformed all other standard classifiers, achieving an average accuracy rate of up to 94.4% in the classification of subjects with ES and PNES, respectively. To analyze the interpretability of the neural network, an information theoretical approach based on PE was proposed. To the best of our knowledge, this is the first application of PE as a measure of interpretability of deep neural networks.

The list of original contributions of the present study can be outlined as follows:

- Development of a data-driven DL pipeline based on CNN and wavelet decomposition for interictal EEG discrimination of ES vs. PNES subjects;
- Development of a information theoretical approach based on PE to perform the interpretability analysis of DL models;
- Development of a system with potential for clinical deployment in the real-world for early or difficult diagnosis.

The remainder of this paper is organized as follows: Section 2 presents the related literature review; Section 3 describes the experimental data, introduces the chosen methodology and itemizes the proposed CNN architecture; Section 4 illustrates the achieved experimental results of CNN and a comparison with standard classifiers; Section 5 and Section 6 address the discussion and conclusions, respectively.

## 2. Related Works

Several EEG-based classification algorithms have been employed to aid the diagnosis among different neurological conditions by using state-of-the-art ML algorithms (i.e., LDA, SVM, ANNs) [9,10,11,12,13,14]. In particular, ML approaches for EEG analysis to early diagnose ES have attracted a lot of interest from the scientific community in recent years [12,15,16]. Rasheed et al. [12] have provided an overview of application of ML methods for predicting ES and [17] have exposed the major issues related to methodology of ES prediction. Varone et al. [15] compared different machine learning algorithms, using the power spectral density of the EEG traces of healthy subjects and with PNES, obtaining a considerable percentage of accuracy. Clarke et al. [16] reports on a deep learning algorithm for computer-assisted EEG review with mean sensitivity of >95% and corresponding mean false positive rate of 1 detection per minute. Ahmadi et al. [18] have classified ES and PNES using the imperialist competitive algorithm on EEG including ictal recordings finding that spectral entropy and Rényi entropy were the most important EEG features, with good classification results using support vector machine (SVM).

Furthermore, PE has been widely employed to directly analyze the temporal information contained in the time series and the abnormalities of brain activity in patients with different neurological conditions [19,20]. Yan et al. [21] proposed an PE network-based algorithm, able to estimate the complexity of EEG signals of control subjects and epileptic patients. They have found lower PE values on EEG signals of epileptic patient compared to control. Li et al. [22] applied PE for predictability analysis of absence seizures, they detect pre-seizure state in 169 out of 314 seizures from 28 rats successfully with an average anticipation time of 4.9 s. Morabito et al. [23] proposed Multivariate Multi-Scale PE to distinguish among the brain states related to Alzheimer’s disease patients and Mild Cognitive Impaired subjects from normal healthy. Its proposal can be used as a complementary synthetic biomarker of the various effects of these diseases on EEG. Mammone et al. [24] introduced the permutation Rényi entropy (PEr) to discriminate interictal states from ictal states in absence seizure EEG. They demonstrated that PEr outperformed PE in their classification, but they still reputed PE a helpful tool to disclose other abnormalities of cerebral electric activity not revealed by conventional EEG recordings [20]. Other uses of PE has been exploited successfully in other areas [25,26,27].

## 3. Materials and Methods

Interictal EEGs were collected from two groups of subjects: patients with new-onset, clinically diagnosed ES, and patients with video-EEG diagnosed PNES. In particular, ES were diagnosed on a clinical basis only, while PNES were diagnosed with the support of video-EEG showing a typical episode (which occurred either spontaneously or following suggestion maneuvers) in the absence of epileptiform EEG activity. Inclusion criteria were: normal interictal EEG at visual inspection, willingness to participate and to give informed consent. Exclusion criteria was the chronic assumption of psychotropic drugs at the time of registration. The EEGs analyzed in this work were collected from the Regional Epilepsy Centre, Great Metropolitan Hospital of Reggio Calabria, University of Catanzaro, Italy. Only EEG recordings free from artifacts were considered.

### 3.1. EEG Data Acquisition

EEGs were acquired by means of Micromed Brain Quick system (Micromed SpA, Mogliano Veneto, Italy) with a sampling rate of 512 Hz, high-pass filter at 0.5 Hz, low-pass filter at 70 Hz, plus a 50 Hz notch filter with a slope of 12 dB/Oct. The EEG signals were acquired using a montage with the following channel layout: Fp1, Fp2, F3, F4, C3, C4, P3, P4, O1, O2, F7, F8, T3, T4, T5, T6, Fz, Cz, Pz and reference in G2 (located between electrodes Fz and Cz). The electrode skin impedance values were kept below 5 KΩ. The EEG data were recorded in a resting condition for 20 min.

All the experiments were conducted in a silent and softly lit room with the subject seated in a handy chair. The subject received information and instruction about the diagnostic setup [28]. After acquisition, EEGs were down-sampled to 256 Hz, segmented into 20 min long records, filtered at 0.5 Hz–32 Hz and stored in the American Standard Code for Information Interchange (ASCII) format for further processing. The EEG recordings were later visually reviewed by experts in order to remove the parts affected by artifacts.

### 3.2. EEG Data Processing

**Figure 1:**
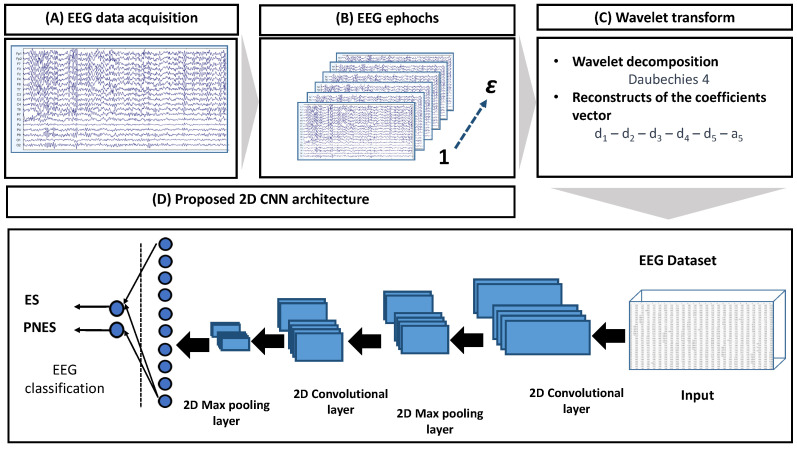
The EEG was recorded and stored on a computer. Therefore, the EEG was cleaned of artifacts, filtered and split into ε non-overlapping epochs of 2 s each. For every ${EEGepoch}^{\varepsilon }$ (ε=1,2,…,214), the wavelet decomposition over each channel was estimated, and subsequently, the sub-bands reconstruction was performed. The database obtained was the input of a convolutional neural network that included 2 convolutional layers (+ReLu activation layer); 2 max pooling layers; 2 fully connected layers, separated by a dropout layer; and a sigmoid layer which performs the classification tasks (two ways: ES vs. PNES).

The flowchart of the proposed method is represented in Figure 1. It includes the following stages: (A) acquisition of the 19-channels EEG recording, EEG signal artefactual reduction (through the labels that have been reported by clinicians during the visual inspection phase) and filtering (Butterworth, 3rd order); (B) partitioning of the EEG signals of a subject into ε non-overlapping epochs of 2 s (the window length was empirically chosen after several experimental tests using an iterative approach); (C) wavelet decomposition with details $d_{1},d_{2},d_{3},d_{4},d_{5}$ and approximation $a_{5}$, i.e., in total 6 sub-bands (chosen through experimental tests), on each channel, on the εth EEG epoch under analysis ( ε = 1, 2, …, 214).

The size of the εth epoch was 19 × 512 × 6. Considering εth (ε = 1, 2, …, 214 is the number of the epoch of all 36 subjects) epochs, we obtained a 4D matrix of ε × 19 × 512 × 6 for each subject. The overall dataset was therefore composed of 7704 × 19 × 512 × 6 ((#epochs × #subjects) × #channels × #samples × #sub-bands); (D) the dataset was used as input to a CNN characterized by 2 convolution layers, 2 max pooling layers and 2 fully connected layers, followed by a sigmoid layer, which performed the 2-way (ES vs. PNES) classification. The leave-one-out cross-validation (LOOCV) [29] was applied. Considering all the epochs of the EEG of a subject, the EEG (and consequently the subject) was assigned to the class with the highest percentage of epochs classified by the network as belonging to that class (either ES or PNES).

#### 3.2.1. Wavelet Transform

Wavelet transform is a time–frequency domain method that allows multi-resolution analysis. It is based on a short wave of limited duration and energy which dilates and shifts along the signal, thereby computing the wavelet coefficients [30,31]. At first, the mother wavelet function (a reference wavelet) is shifted continuously along the time scale to obtain a set of coefficients in time. Subsequently, the wavelet is dilated to a different width and then normalized in order to estimate the corresponding group of coefficients [32]. There are three types of wavelet transformations: Discrete Wavelet Transform (DWT), Continuous Wavelet Transform (CWT) and Wavelet Packet Decomposition (WPD). Of these, the most widely used for EEG analysis in the context of epilepsy is DWT [33] because it captures transient features and accurately localizes them both in time and frequency content [34]. The DWT wavelet function can be written as:

(1) $$\Psi_{j,k}\left(t\right)=2^{-\frac{j}{2}}\Psi \left(2^{-j}t-k\right)$$

where *k* is the shift parameter, *j* is the resolution level; the greater the value of *j*, the smaller the frequency. The discrete wavelet decomposition coefficient $W_{jk}$ can be written as:

(2) $$\;W_{jk}=2^{-\frac{j}{2}}\sum_{n}x\left(n\right)\Psi \left(2^{-j}n-k\right)$$

from the discrete wavelet decomposition coefficient $W_{jk}$ the original signal *x*(*t*) can be reconstructed:

(3) $$\;x\left(t\right)=\frac{1}{C}\sum_{j}\sum_{k}W_{jk}\Psi_{j,k}\left(t\right)$$

**Figure 2:**
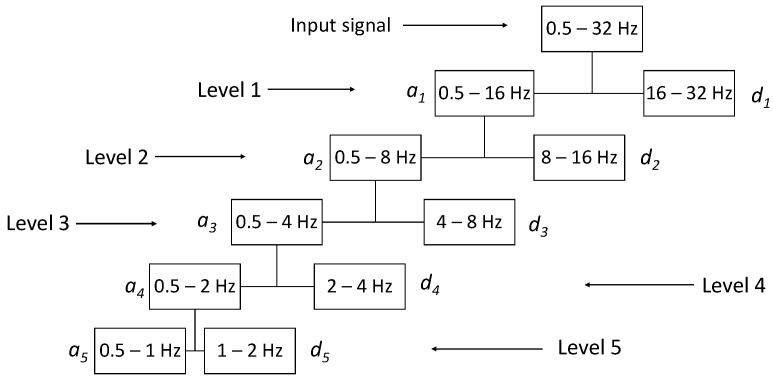
Wavelet transform decomposition tree of the EEG signal, based on the Mallat algorithm.

The wavelet decomposition of a signal can be carried out through filtering in a cascade of two band-pass filters, as stated by the Mallat algorithm [35,36]. The detail coefficient, $c_{n}$ and the approximation coefficient, $a_{n}$, are computed by quadrature mirror filters, and the signal is reconstructed following the scheme of filters known in the literature. A graphic representation of a DWT signal is shown in Figure 2.

**Figure 3:**
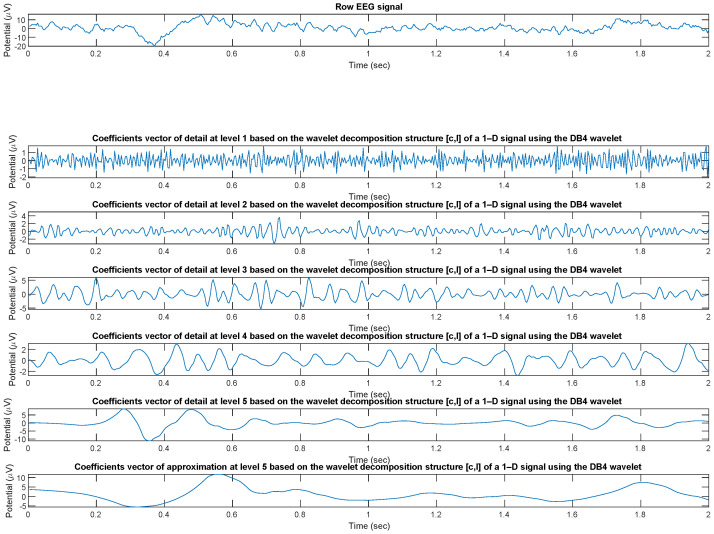
Coefficients vectors of detail and approximation coefficients based on the DB4 wavelet decomposition structure [c,l] of a sample EEG signals.

The wavelet analysis (decomposition) of the EEG signal was performed through the *wavedec* [37] function implemented in MATLAB which returns the wavelet decomposition of the 1-D signal *x(t)* of every *i*th channel (with i = 1, 2, …, 19) of every εth epoch (with ε = 1, 2, …, 214) of every *m*th patient (with m = 1, 2, …, 36)) at level n (with n = 1, 2, …, 5) of details ($d_{n}$) and 5 of approximation ($a_{5}$) using the Daubechies 4 (DB4) wavelet. Other mother wavelets can be used, as appropriate for experimental biosignals [38]. The output decomposition structure consists of the wavelet decomposition vector c and the bookkeeping vector l, which contains the number of coefficients by level. Subsequently, using the *wrcoef* [39] function, also integrated in MATLAB, the coefficients vector of type based on the wavelet decomposition structure [c,l] of a 1-D signal (*wavedec*) using the wavelet DB4 was reconstructed (Figure 3).

Therefore, the signal processing pipeline can be summarized in two blocks: wavelet decomposition (energy of details $d_{1},d_{2},d_{3},d_{4},d_{5}$ and approximation coefficient $a_{5}$, for a total of 6 bands) and reconstructions from the coefficients vector. Figure 3 shows the reconstructed signal in the respective sub-bands.

The original signal *x*(*t*) can be reconstructed directly by the approximation signal plus the detail signals as specified in the following equation:

(4) $$\;x\left(t\right)=a_{5}+d_{5}+d_{4}+d_{3}+d_{2}+d_{1}$$

#### 3.2.2. Convolutional Neural Network

In the last decades, Deep Learning or Deep Neural Networks have been regarded as powerful tools as they are able to handling a huge amount of data. One of the most popular deep neural networks is the CNN. It has already surpassed the performance of classical pattern recognition methods in several fields and is expected to be increasingly used. The most important improvement of CNN with respect to previous techniques is the reduction of the number of parameters in artificial neural network (ANN). This allowed the analysis of complex and large data (such as biosignals and images) that previously was impossible to deal. CNN has multiple layers, including convolutional layer, non-linearity layer, pooling layer and fully-connected layer [40]. The CNN includes two big building blocks: a feature extractor and an ANN block. The first one characterizes the CNN network. It automatically extracts the features from the raw signal and consists of convolution, activation and pooling layers. The ANN is a fully connected multi-layer neural network widely applied in many classifiers based on neural networks (e.g., MLP). The task of this block is to perform the classification exploiting the previously learned features.

In detail, the *convolution layer* performs the convolution operation formulated as:

(5) $$\;Y_{j}=\sum_{}X_{i}*K_{j}+B_{j}$$

The output $Y_{j}$ is a feature map of every $K_{j}$ filter convolved (∗) with a local region of $X_{i}$ (called receptive field) and the bias $B_{j}$ added. The size of $Y_{j}$ depends on the padding parameter and the size and the stride of the filters; the amount depends on the number of filters. Each filter moves along the input with a specific step size (sharing the same weights), estimating C feature maps (with C = number of filters). The convolution layer is followed by an *activation layer*, a nonlinear transfer function that could be *sigmoid*, *hyperbolic tangent* or *rectified linear units*. The last one was considered better in terms of generalization and learning time for CNN by recent studies [41,42]. In the *pooling layer*, the extracted features maps are downsampled through a max or average pooling layer. The filter scans the features map of the input and calculates the maximum or the average of each sub-region being analyzed, returning a map of reduced size. The latter building blocks (ANN) consists of one or more fully connected layers. The output of ANN perform the discrimination task.

#### 3.2.3. Proposed Architecture

The proposed CNN architecture includes 2 convolutional layers (+ReLu activation function), 2 max pooling layers, 2 fully connected layers and a sigmoid layer which performs the classification tasks (two ways: ES vs. PNES). The sizing of the network (number of levels, number and size of filters, etc.) was chosen empirically after several experimental tests using an iterative approach aimed at improving network performance and automatically extracting features. It is designed to accept the fixed sizes matrix of c × s × f (where c = 19, number of EEG channels; s = 512, number of signal samples 256 Hz ∗ 2 s; f = 6, number of sub-bands of wavelet decomposition). The convolutional layer ($Conv_{1}$) has 16 learnable filters, each sized 1 × 6. Every filter convolves with each temporal input representation. It also has "SAME" padding and a stride of 1 × 2. The layer’s outputs with the same spatial 1st dimensions and half 2nd dimensions as its inputs generates. $Conv_{1}$ is followed firstly by the ReLu and then by the max pooling layer ($MaxPool_{1}$) which reduces the features maps size from 19 × 256 × 16 to 19 × 128 × 16 by using 1 × 2 filters with a stride s = 1 × 2. The convolutional layer ($Conv_{2}$) has 32 learnable filters sized 1 × 3 with a strides 1 × 2. Furthermore, $Conv_{2}$ is followed firstly by the ReLu and then by the max pooling layer ($MaxPool_{2}$) which reduces the features maps size from 19 × 64 × 32 to 19 × 32 × 32 by using 1 × 2 filters with a stride s = 1 × 2. These levels extract the most relevant features automatically. The details on the number of parameters used are shown in Table 1.

The features extracted are flattened (Flatten layer) and are used as an input of a ($Dense_{1}$) layer with 32 hidden neurons. It is followed by a Dropout layer (of 0.3, in order to improve generalization and avoid overfitting) which separates from $Dense_{2}$ with 16 hidden neurons. The network ends with a sigmoid layer ($Dense_{3}$) to estimate the class predictions in binary classification. If the network’s output is less than 0.5 the epoch is considered of PNES class (labeled as 0); and if the output of the network is greater than 0.5, then the epoch is considered of ES class (labeled as 1). The proposed CNN was made in Python, using Keras [43] with Tensorflow backend, and trained using the default parameters of the adaptive moment estimation (ADAM) optimizer [44] for 10 iterations and with a batch size of 107 until the crossentropy function converged.

### 3.3. Performance Metrics of Classification

A balanced dataset of 7704 EEG epochs (3852 related to 18 ES subjects and 3852 related to 18 PNES subjects) was used to test the proposed DL pipeline of EEG classification that was composed of two main building blocks: wavelet decomposition, which extracted 6 sub-bands from EEG raw data, and the convolutional neural network, which performed the classification task. The aim was to carry out an overall patient-based classification on the basis of how their epochs have been labeled by the trained network. For the patient-based classification, given a subject, if the number of epochs labelled by the network as a specific class is larger than 50%, then the subject is assigned to that class. In order to estimate a better true prediction error, tune parameters estimation and to prevent overfitting of models, a k-fold cross-validation data resampling methods is used. We chosen a special case of k-fold cross-validation, called leave-one-out cross-validation (LOOCV). Here, each subject serves, in turn, as hold-out case for the test set. The available learning set is partitioned into 36 (number of subject) disjoint subsets of equal size [45,46]. Specifically, the CNN was iteratively trained using the whole data set and leaving out the epochs of a single subject at a time. Therefore, 36 models were trained and the instances (i.e., epochs of $Sb_{j}$) left-out represented the test set of the *j*th network. The classification performance were evaluated using the following standard metrics:

(6) $$\;ACCURACY=\frac{TP+TN}{TP+TN+FP+FN}\;$$

(7) $$\;PRECISION=\frac{TP}{TP+FP}\;$$

(8) $$\;RECALL=\frac{TP}{TP+FN}\;$$

(9) $$\;F-measure=2*\frac{PRECISION*RECALL}{PRECISION+RECALL}\;$$

(10) $$\;Cohen^{\prime }s-kappa=\frac{2*(TP*TN-FN*FP)}{\left(TP+FP)*(FP+TN)+(TP+FN)*(FN+TN\right)}\;$$

where *TP*, *TN*, *FP* and *FN* represent the true positive, true negative, false positive and false negative, respectively. Specifically, *TP* and *TN* are the numbers of PNES and ES subjects classified correctly; *FP* is the number of ES subjects incorrectly classified as PNES and vice versa, *FN* is the number of PNES subjects misclassified as ES subjects.

### 3.4. Comparison with Standard Classifiers

The proposed CNN was also compared with standard classifiers to evaluate the specific efficiency of the DL approach. In detail, the multi-layer percerptron (MLP), support vector machine with a Gaussian radial basis function kernel ($SVM_{rbf}$), linear discriminant analysis (LDA) and quadratic discriminant analysis (QDA) were trained on handcrafted features extracted from wavelet sub-bands. Specifically, for each epoch of the *m*th patient (m = 1, 2 …, 36), six characteristics discriminating from the six EEG signal sub-bands were calculated: Min, Max, Energy, Mean, Std and Skewness. The last three features (Mean, Std and Skewness) were chosen in agreement with Gasparini et al. [47]. Additional information was obtained including Min, Max and Energy, according to Hamad et al. [33].

The resulting handcrafted feature vectors included six features for each electrode of the six sub-bands. The feature vector was sized 684 (6 × 6 × 19) for each εth epoch. The overall dataset composed of 7704 × 684 (#epochs × #feature vector) was used as input for the standard classifiers to identify EEG patterns of ES and PNES. Six classifiers were trained and tested: $MLP_{1}$ with 1 layer of 300 neurons; $MLP_{2}$ with two layers of 300 and 50 neurons, respectively; $MLP_{3}$ with three layers of 300, 100 and 50 neurons, respectively. All MLP classifiers ended with a softmax output layer to carry out the binary classification (ES vs. PNES), and each was trained using the default parameter of adaptive moment estimation (ADAM) optimizer [44] for 10 iterations with a batch size of 214 until the crossentropy function converged. The $SVM_{rbf}$ was trained using the Gaussian kernel radial basis function with regularization parameter (gamma) of 0.001. The LDA was trained using the default “singular value decomposition (SVD)” solver without using shrinkage. The QDA, at last, was trained using the default hyperparameter for regularization, which is “reg_param”, of 0.0. The topology of the classifiers was chosen empirically after several experimental tests.

### 3.5. Permutation Entropy Based Interpretability of Proposed Architecture

The evaluation of the classification efficiency in deep neural networks is generally done by analyzing the inputs and the outputs. For performing interpretability analysis of the behavior of the proposed CNN, we extracted the feature maps in the intermediate layers ($Conv_{1}$, $Conv_{2}$) and compared them with the input. The goal was to inspect the separability of the latent features in the intermediate transformation. From an information theoretical perspective, by passing through the various successive stages of the CNN, the input vector meliorates its discrimination capability by improving the mutual information (MI) between the presently available vector/image and the corresponding label. This progressive modification of the MI corresponds to a reduction in the uncertainty measurable by suitable entropic parameters. Permutation entropy (PE) [48] was used here for the quantitative comparison. PE is a natural complexity measure for time series that can be calculated for arbitrary real-world time series. PE computation is extremely fast and robust to noise and outliers. The PE of a signal *x* is defined as:

(11) $$\;PE\left(n\right)=-\sum p\left(\pi \right)log_{2}\left(\pi \right)\;$$

where the sum runs over all *n*! permutations π of order *n* [49]. Referring to $\pi_{n}$ as the permutations of time series, the relative frequency of each π is obtained by counting the number of times π is found in the time series divided by the total number of sequences. This is the information contained in comparing n consecutive values of the time series. In particular, PE was computed by using AntroPy [49], a Python 3 package that provides several time-efficient algorithms for computing the complexity of time-series.

#### Statistical Test

The separability of the input data, and the latent features in $Conv_{1}$ and $Conv_{2}$ layers in terms of PE, were statistically quantified by using the Wilcoxon rank-sum test. It tests the null hypothesis that two sets of measurements are drawn from the same distribution. The other hypothesis is that values in one sample are stochastically larger than the values in the other sample [50]. The Wilcoxon rank-sum test was preferred to the two-sample *t*-test because it is much less sensitive to outliers [51]. Furthermore, to provide statistical support for the analysis and comparison of the proposed CNN architecture and standard classifiers’ results, the posthoc Friedman-Nemenyi test was performed. In particular, the Friedman test is a non-parametric test used to determine whether or not there is a statistically significant difference between the accuracy of classifiers when the same subjects show up in each classifier [52,53]. If the Friedman test *p*-value was statistically significant, the post hoc Nemenyi test to determine exactly which results are different was to be executed [54,55].

## 4. Results

### 4.1. Performance of the Proposed Architecture and Comparisons with Standard Classifiers

The experimental data available consisted of 18 patients with ES (mean age 47.9 years, 12 males, 6 females) and 18 patients with PNES (mean age 27.3 years, 2 males, 16 females). The experimental data were balanced, and there is no evidence to suggest that age-related changes in EEG could have affected the results [56,57].

The proposed CNN achieved very good values in each test scenario, reporting accuracy rates up to 94.4% for the patient-based classification. Encouraging values of recall, precision F-measure and Cohen’s kappa were observed. Table 2 outlines the values of the patient-based test results of CNN in comparison with $MLP_{1}$, $MLP_{2}$, $MLP_{3}$, $SVM_{rbf}$, LDA and QDA classifiers.

**Figure 4:**
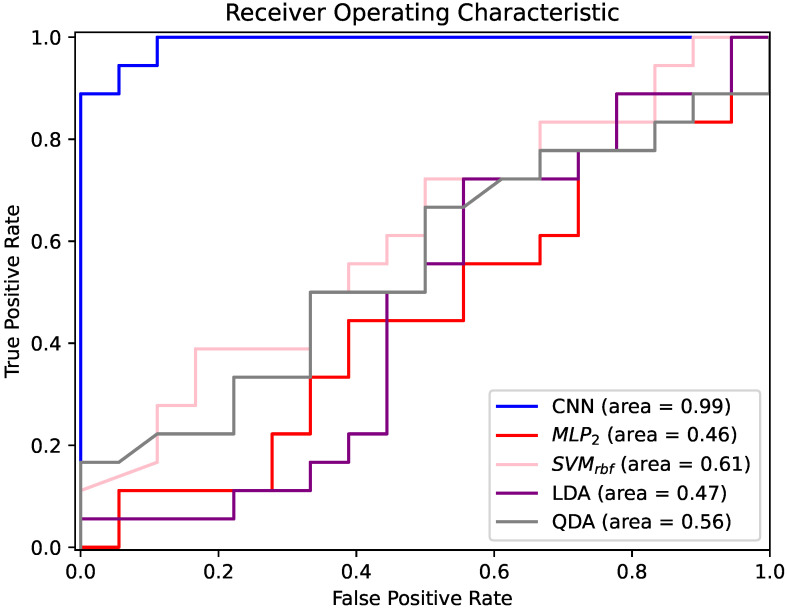
ROC curves of CNN, $MLP_{2}$, $SVM_{rbf}$, LDA and QDA classifiers for ES vs. PNES classification. Features automatically extracted from sub-bands were used as input for the CNN, and handcrafted features manually extracted from sub-bands were used as inputs for $MLP_{2}$, $SVM_{rbf}$, LDA and QDA. $MLP_{1}$ and $MLP_{3}$ present similar trends to $MLP_{2}$, which, however, had higher accuracy. Therefore, for better visual understanding, they were not included in the graph.

The good network performance was confirmed by the analysis of the area under the curve (AUC) of the receiver operating curve (ROC). As shown in Figure 4, the patient-based classification had an AUC of 0.99, indicating the excellent discrimination properties of the CNN.

Different training options were evaluated for CNN, $MLP_{1}$, $MLP_{2}$ and $MLP_{3}$: varying the learning rate α, the rate of decay of the first moment $\beta_{1}$ and the rate of decay of the second moment $\beta_{2}$. It was observed that the best results were obtained with: learning rate $\alpha =10^{-2}$, first moment decay rate $\beta_{1}$ = 0.9 and second moment decay rate $\beta_{2}$ = 0.999 according to the practical recommendations reported in [44,58]. The SVM was evaluated using different kernels: polynomial kernel, gaussian kernel, radial basis function (RBF), Laplace RBF kernel and sigmoid kernel. It was observed that the best results were obtained using the RBF kernel with a regularization parameter (gamma) of 0.001. Furthermore, for the valued LDA, different solvers were used: least squares solution (lsqr), eigenvalue decomposition (eigen) and the default singular value decomposition (svd) solver that was chosen with a regularization parameter (gamma) of 0.001 because it obtained the best accuracy. The best results for the QDA training were obtained using the default hyperparameter for regularization, which is “reg_param”, of 0.0.

Average training and test times were recorded to evaluate the computational cost and the latency introduced by the classifiers, in order to evaluate possible use in the clinical diagnostic routine. The total processing time of the proposed CNN for patient-based classification was 1624 s (27 min). Of note, the total processing time included all the 36 iterations (same for all classifiers). Each iteration had average durations of 45 s and 2 s for training and testing, respectively. Therefore, despite the long training times, the time of inference introduced by the network is only 2 s.

The total processing time of the $MLP_{1}$ for patient-based classification was 854 s (14 min). Each iteration had average durations of 19 s or 4 s for training and test, respectively.

The total processing time of the $MLP_{2}$ for patient-based classification was 661 s (11 min). Each iteration had average durations of 15 s and 3 s for training and testing, respectively.

The total processing time of the $MLP_{3}$ for patient-based classification was 443 s (7 min). Each iteration had average durations of 5 s and 2 s for training and testing, respectively.

The total processing time of the $SVM_{rbf}$ for patient-based classification was 792 s (13 min). Each iteration had average durations of 12 s and 10 s for training and testing, respectively.

The total processing time of the LDA for patient-based classification was 47 s. Each iteration had average durations of 1.3 s and 0.004 s for training and testing, respectively.

The total processing time of the QDA for patient-based classification was 52 s. Each iteration had average durations of 1.4 s and 0.02 s for training and testing, respectively.

Of note, all performances were obtained using Intel(R) Xeon(R) GPU NVIDIA Tesla K40c with 12 GB RAM.

### 4.2. Interpretability of the Proposed Deep Learning Model and Statistical Testing

**Figure 5:**
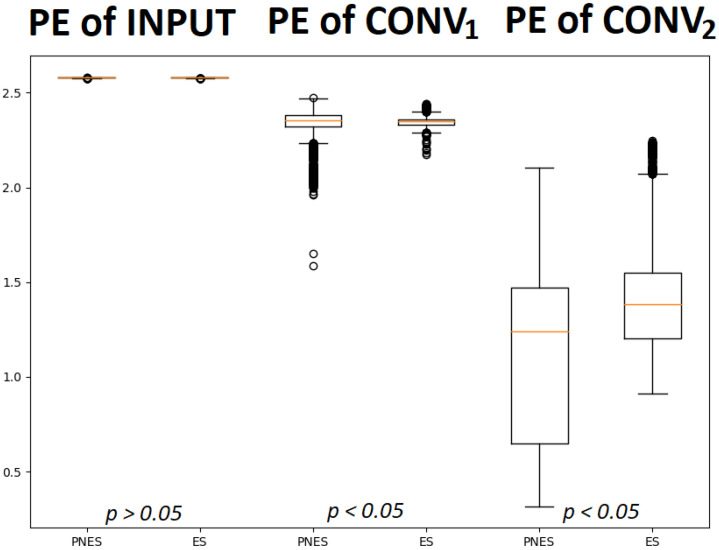
The box plots describe the permutation entropy distribution of the class of subjects with PNES and ES referred to input layer (1st and 2nd box plots), $Conv_{1}$ layer (3rd and 4th box plots) and $Conv_{2}$ layer (5th and 6th box plots). It is worth noting that the PE of input did not allow significant discrimination between the two classes. Instead, statistically quantified differences, with a *p*-value < 0.05, were found between the PE of PNES and ES subject in $Conv_{1}$ and $Conv_{2}$.

Figure 5 shows the difference between the two classes in terms of PE in the successive levels of the proposed scheme described above.

**Figure 6:**
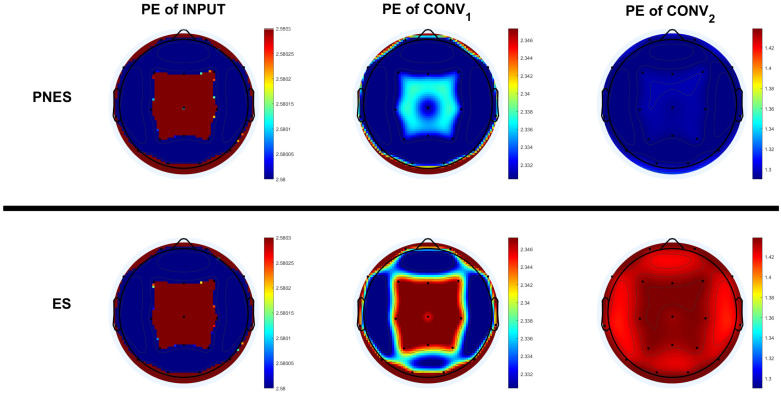
The topo-plots describe the permutation entropy distributions of the channels of the class of subjects with PNES and ES referred to input layer (**topo-plots on the left**), $Conv_{1}$ layer (**topo-plots on the middle**) and $Conv_{2}$ layer (**topo-plots on the right**). For the input, there are no significant differences between the two classes. Instead, differences, in frontal and parietal regions, were found between the PE of PNES and ES subjects in the $Conv_{1}$, and differences in all channels in the $Conv_{2}$.

We obtained the *p*-value corresponding to the Wilcoxon rank-sum test statistic. The results show no statistically significant differences for the PE of input values at first, though the differences became statistically significant deeper in the network. The separability between the two groups/classes increased with the depth of the network. The average values of PE of the latent features of each channel were then analyzed. Figure 6 shows the differences between the two classes. The input did not show a difference in PE values between the two classes. Instead, a difference in PE among the channels was noted, in particular, in frontal and parietal regions.

The features extracted from the EEG in the central and parietal areas of the subjects with PNES have a lower PE compared to the subjects with ES in the $Conv_{1}$. More distributed differences of PE have been found in the features of $Conv_{2}$.

To perform multiple comparisons between our proposed architecture and the standard classifiers considered in this study, we performed the Friedman test to highlight statistical differences among accuracy results, and the Bonferroni and Nemenyi post hoc tests to discover which algorithms are statisticaly distinctive among the comparisons performed. Friedman’s test showed a *p*-value = 0.0002, less than 0.005 so statistically significant. That is, we have sufficient evidence to assert that the type of classifiers used conduct to statistically significant differences in accuracy results. Subsequently, it was possible to perform the post hoc Nemenyi test to determine exactly which classifier is statistically significant. The Nemeyi post hoc test returns the *p*-values for each pairwise comparison of classifiers. From the output, at *p*-value < 0.05, we have been able to affirm that only the proposed CNN obtained statistically significantly different accuracy than all other classifiers. In detail, proposed CNN vs. $MLP_{1}$: *p*-value = 0.001; CNN vs. $MLP_{2}$: *p*-value = 0.004; CNN vs. $MLP_{3}$: *p*-value = 0.001; CNN vs. $SVM_{rbf}$: *p*-value = 0.04; CNN vs. LDA: *p*-value = 0.004; CNN vs. QDA: *p*-value = 0.02.

## 5. Discussion

In this paper, we provided a DL tool capable of discriminating with good reliability between two classes of subjects with ES or PNES. In particular, this discrimination is realized by a DL algorithm (CNN) analyzing latent features extracted from time-frequency sub-bands of noninvasive interictal scalp EEG recordings. This CNN was compared with the most used standard classifiers, and it showed better performance compared to other techniques, thereby proving to well exploit the strong non-linearity of the incoming data. Note that the proposed CNN performed the classification using the raw data, and it was the only one to obtain acceptable results, whereas the standard classifiers used the features described in Section 3.4. No standard classifiers achieved acceptable accuracy. $MLP_{1}$, $MLP_{2}$ and $MLP_{3}$ obtained comparable results despite the variation in the number of parameters and depth of the network. Furthermore, $SVM_{rbf}$, LDA and QDA also did not show any great improvement during the many experimental tests. This can be attested to by the reduction of the information of interest contained in the raw data in the transition to handcrafted features. Thus, the manual extraction of the features, in addition to being time consuming, cannot capture the latent information that can be used for the classification of the two classes. Therefore, despite DL being much more complex in terms of design and thus more computationally intensive, as outlined in Section 4.1, it allows one to bypass the time-consuming and expensive handcrafted feature extraction process, and it is able to learn representations of data with multiple levels of abstraction [59]. DL overcomes the limits of standard learning algorithms, allowing more detailed analyses thanks to the extraction of multidimensional latent features. DL often suffers from a lack of interpretability. Keeping track of all non-linear transformations and the large number of free parameters within the network is indeed very hard. The progress of neural networks has not been followed by a complete understanding of the highly non-linear transformations, so their use in critical sectors such as the health, where interpretation of the actions taken is requested, has not increased greatly. A deeper understanding and the appropriate level of trust will lead to increasing adoption of this technology in critical applications, such as the present challenging PNES vs. ES classification. The explanability of DL, indeed, is a powerful tool for detecting flaws in models and biases in the data; for verifying predictions; for improving models; and finally, for gaining new insights into the problem at hand, and will become of fundamental importance for applications in the medical domain where wrong decisions of a system can be very harmful. Therefore, interpretability DL has attracted much interest from the data scientist community [60,61,62].

In the literature there are several promising results achieved by AI algorithms on neurological conditions [9,10,63,64]. Only two works have presented classification studies differentiating ES and PNES [18,65], both based on ML. The first study [18] performed a classification of 20 epilepsy and 20 PNES patients by using the imperialist competitive algorithm for feature extraction, achieving an accuracy higher than 90%. However, they used the EEGs including periods of seizures: this was likely to result in a significant difference between the two groups. In another study of the same group [65], the authors studied the interictal EEGs of five subjects with ES and five subjects with PNES by feature extraction for automatic classification and functional brain network analysis. The accuracy was found to be around 80% when the classification was computed based on the microstate features extracted from the beta-bands. This relatively low value of accuracy have resulted from the small sample size and the complexity of classification task. This supports the hypothesis that these interictal EEG classifications are still an open challenge.

With the aim of studying the behavior of the neural network, we analyzed the feature maps, which are the results of the latent transformations of the input. The better data separability deeper in the network testifies to good learning of distinctive features. Therefore, the convolutional block was able to identify distinctive features without the need for known biomarkers, but with a data-driven approach. The study of PE throughout the depth of the proposed deep neural network allowed us to interpret the behavior of the model, without explaining, however, why the model behaves the way it does. PE differences were also found between the central and parietal areas, in agreement with other studies [66,67,68] claiming that these areas play an important role in liability for seizures in ES subjects.

## 6. Conclusions

In conclusion, our proposed pipeline has shown very high accuracy for the fully automatic supervised classification of the two classes of analyzed subjects. Reviewing EEG data by means of DL algorithms would save time and resources, thereby allowing a larger number of people to obtain reference standard monitoring and also providing standardized support for physicians in therapeutic settings. Nevertheless, the DL approach is not widely used in the clinic, despite the increasing number of DL algorithms being published, but our interpretability analysis through PE could increase confidence in these algorithms and foster regulatory approval to discriminate ES and PNES. To the best of our knowledge, this study is the first application of PE as a measure of the interpretability of DL networks. Therefore, this pipeline is fast to apply, and it may be used in day-to-day hospital care during routine analyses, to make early and effective diagnosis potentially available on a large scale. This should encourage the use and trust of DL methods to support clinicians in future diagnostic applications.

## Figures and Tables

**Table 1:** Total number of learnable parameters for the proposed CNN architecture that includes 2 convolutional layers (+ReLu), 2 max pooling layers, 2 fully connected layers, one dropout layer and a sigmoid layer which performs the classification tasks.

Layer Name	Output Shape	Parameters
Input	19 × 512 × 6	
$Conv_{1}$	19 × 256 × 16	592
$MaxPool_{1}$	19 × 128 × 16	
$Conv_{2}$	19 × 64 × 32	1.568
$MaxPool_{2}$	19 × 32 × 32	
Flatten	19456	
$Dense_{1}$	32	622.624
Dropout	32	
$Dense_{2}$	16	528
$Dense_{3}$	1	17
Total		*625.329*

**Table 2:** ES andPNES subject classification performances (accuracy, precision, recall, F-measure, Cohen’s kappa) evaluated with patient-based classification with the proposed CNN and standard classifiers.

ES vs. PNES					
**Classifier**	**Accuracy**	**Precision**	**Recall**	**F-Measure**	**Cohen’s Kappa**
CNN	94.4%	89.9%	100%	94.7%	88.8%
$MLP_{1}$	38.9%	41.7%	55.6%	47.6%	−22.2%
$MLP_{2}$	47.2%	47.4%	50.0%	48.6%	−5.6%
$MLP_{3}$	44.4%	44.4%	44.4%	44.4%	−11.1%
SVM	58.3%	57.1%	66.7%	61.5%	16.7%
LDA	55.6%	54.5%	66.7%	60.0%	11.1%
QDA	55.6%	54.5%	66.7%	60.0%	11.1%

## Data Availability

Not applicable.
